# Management of diplopia

**DOI:** 10.22336/rjo.2017.31

**Published:** 2017

**Authors:** Daniela Adriana Iliescu, Cristina Mihaela Timaru, Nicolae Alexe, Elena Gosav, Algerino De Simone, Mehdi Batras, Cornel Stefan

**Affiliations:** * Ophthalmology Department, “Dr. Carol Davila” Central Military University Emergency Hospital, Bucharest, Romania

**Keywords:** diplopia, binocular vision, strabismus

## Abstract

Diplopia (seeing double) is an ophthalmologic complaint found mainly in elder patients. It can have both ocular and neurological causes. A careful history and clinical examination must detail the type of diplopia (monocular/ binocular), onset, and progression, associated and relieving factors. In case of monocular diplopia, refraction and biomicroscopic examination of the ocular media are mandatory. The cause of ocular misalignment for binocular diplopia must be determined and life-threatening conditions (such as posterior communicating artery aneurysm) must imply an immediate treatment. Management and treatment is always according to the specific cause of diplopia.

## Introduction

Diplopia - simultaneous perception of two images of a single object or seeing double is a common symptom identified in ophthalmological and neurological patients. It has many underlying causes. An efficient management implies an accurate diagnosis that can be made with a detailed history and a careful clinical examination. The assessment of the patient’s perception of diplopia must exclude other symptoms that can be misunderstood by the patient, such as image distortion, vision field defects, after images, hemianopia. The first aspect that must be determined is whether the diplopia is monocular or binocular. The difference is made by covering one eye. If the diplopia persists after one eye occlusion, then the diplopia is monocular. If the diplopia disappears after the eye is occluded, then it is binocular and an extensive investigation must make the differential diagnosis between multiple etiologies that can cause the misalignment of the visual axes [**1**]. 

## Monocular diplopia

Monocular diplopia is often caused by uncorrected refractive errors (astigmatism) and cataract. It is usually due to intraocular pathology that requires a detailed ophthalmological evaluation. Monocular diplopia or polyopia (> 2 images), unilateral or bilateral, are rarely of cerebral cause, determined by primary and secondary visual cortex lesions. Associated symptoms such as decreased visual acuity and haze can appear in diplopia caused by cataract. Macular disorders can associate metamorphopsia (Amsler grid evaluation). Other causes of monocular diplopia include dry eye syndrome, wrong-fitting contact lens, irregular corneal surface, keratoconus, abnormalities of the iris (iridodialysis, polycoria, and large iridotomies), and vitreous opacities. The pinhole test will differentiate between an optical cause and other forms of monocular diplopia. The optical causes can then be treated with glasses, contact lenses, refractive or cataract surgery and artificial tears. 

Physiological diplopia is the phenomenon in which targets that are not in the area of focus, but in front or behind the point of fixation, are seen as double. It is more common in children; in adults, it is usually centrally suppressed. The test of looking at a distant object while fixation is directed to a near target must demonstrate the phenomenon and no other eye movement abnormalities or underlying neurological disorder must be reassured.

## Binocular diplopia

**History of binocular diplopia**

A detailed history of diplopia has a very important role in establishing the diagnosis. The assessment must include the onset, quality, direction, comitancy, variability or fatigability, associated symptoms or diseases. The onset of diplopia is most often sudden, but it can also be gradual, determined by trauma, or spontaneous. While the acute onset is more suggestive of a vascular event but is not very specific, the gradual progression or diplopia that has changed the pattern is more indicative of a compressive lesion. The direction of diplopia can be horizontal, vertical, and oblique. When right and left rectus muscles are affected either by impaired nervous control or by muscle function, the diplopia is horizontal. The most common causes of horizontal diplopia are VIth nerve palsy and internuclear ophthalmoplegia. Horizontal diplopia that appears only after a prolonged near vision is highly pointing towards a convergence insufficiency (most common in patients with Parkinson’s disease). Diplopia is worst (the patient has a maximal separation of images) in the region of action of the paralyzed muscle and the false image (the image that belongs to the eye with the impaired muscle) is always peripherally situated. Vertical diplopia can appear in thyroid eye disease (inferior rectus muscle is most commonly affected), orbital floor fracture, trochlear nerve palsy, supranuclear or infranuclear lesions. Oblique and torsional (tilted image) diplopia are seen in superior and inferior muscle impairment and lateral medullary syndrome. Comitant deviations will be equal in all directions. Usually, comitant deviations do not induce diplopia due to cortical suppression (patients with congenital strabismus). Variations of diplopia during the day, with an exacerbation in the second half, improvement after rest and associated ptosis that is also intermittent are suggestive of myasthenia gravis (autoimmune neuromuscular junction disease) [**2**]. 

**Evaluation for binocular diplopia**

The examination of binocular diplopia must assess the presence or absence of local findings (eyelid position, facial sensation, orbicularis oculi strength, and exophthalmos) and ocular motility. An important displacement of the ocular globe must be determined if resulting from exophthalmos or enophthalmos. Spontaneous (exophthalmos, congestion) or posttraumatic local findings must include tonometry, ultrasound, CT scan, MRI, neurologic and neuro-surgery examination in their evaluation. The alignment of the eyes starts with the observation of the eyes in primary position, upgaze, downgaze, lateral gaze, and cover-uncover test. Cross-cover test will identify the presence of latent deviation (phoria). Hess test, Maddox rod and red glass testing are useful for the measurement of ocular deviation. Parks-Bielschowsky test is used for the identification of the paretic muscle in vertical diplopia. Forced duction test can identify if the restricted movement of the eye is due to mechanical restriction (important to assess in blow out fractures) or agonist muscle weakness. The misalignment of visual axes is most often due to the dysfunction of extraocular muscles.

## Cranial nerve palsies

**Oculomotor nerve palsy**

Complete oculomotor nerve palsy is revealed when superior, inferior, medial recti, oblique inferior and superior levator palpebral are affected giving a dysfunction that will generate ptosis and downgaze with lateral gaze position of the eye. Paralysis of ciliary muscle and pupillary sphincter will associate fixed dilated pupil. Describing the pupil as spared or involved is very important. Pupil sparing will show a normal size and reactivity to light, contrary to the pupillary involvement that indicates mydriasis and hyporesponsiveness to light and accommodation. In case of relative pupillary sparing, the pupil is affected, but more than 0.5 mm and up to 2 mm remains reactive to light. Due to the anatomical placement of the pupillary fiber along the superficial layer of the nerve, pupil involvement is present when there is external compression (especially posterior communicating artery aneurysm). In this case, emergency MRI or CT of the brain is required, followed by catheter angiography. Neuroimaging is not necessary in complete, pupil-sparing oculomotor palsies that appear in older patients over 50 with multiple vascular risk factors (diabetes, hypertension, and atherosclerosis). Moreover, the third nerve palsy is secondary to vascular microinfarction. If after 3 months from third nerve palsy onset, the recovery is not complete, or there are other cranial nerve involvements, neuroimaging must be indicated. If the pupil is dilated and unresponsive but there is no extraocular movement or eyelid impairment, the patient does not present with a third nerve palsy [**3**].

**Trochlear nerve palsy**

Trochlear nerve palsy will impair the function of superior oblique muscle. It causes vertical, oblique, or torsional diplopia that is more pronounced in downgaze. These patients usually develop a contralateral head tilt as a reaction to counteract the vertical diplopia. If ipsilateral oculomotor nerve palsy is also present, testing the superior oblique muscle function can be difficult because the muscle acts as a depressor only if adduction is present. In this situation, if the eye can do intorsion on attempted downgaze, the muscle may have a normal function. Ocular motility testing will show ipsilateral hypertropia that is emphasized on ipsilateral head tilt and contralateral gaze. Thyroid eye disease and myasthenia gravis must be excluded for apparent trochlear nerve palsy. Besides microvascular disease, a frequent cause of trochlear nerve palsy is head trauma. Intermittent diplopia can appear in decompensated congenital trochlear nerve palsy (patients present with long-standing head tilt) or superior oblique myokymia that is a rare monocular disorder, in which high frequency burst will contract the muscle those last seconds and occurs multiple times per day. The fourth cranial nerve has a short course, and, in isolated palsies, is usually not affected by aneurysm, tumors or demyelinating processes [**4**].

**Abducens nerve palsy**

The abducens nerve palsy (has the longest intracranial course) is the most common nerve palsy, followed by trochlear and oculomotor in second and third place. Due to its long path, it is sensitive to direct and indirect lesions. The sixth nerve palsy will generate lateral rectus muscle paresis (esotropia) and horizontal diplopia that is worse at distance. The causes of sixth nerve palsy may include microvascular ischemia that requires neuroimaging (MRI with gadolinium) if there is progression or absence of improvement, and raised intracranial pressure if the dysfunction is transient, frequently bilateral but asymmetric. Raised intracranial pressure requires neuroimaging and sometimes lumbar puncture. Myasthenia gravis and Duane retraction syndrome must be excluded in abducens nerve palsy thyroid eye disease [**5**].

## Convergence insufficiency

Convergence insufficiency is a sensory and neuromuscular disorder of the binocular visual system that causes horizontal diplopia after prolonged near vision. It is present in children and adults, and is of idiopathic cause or can be developed after head trauma. Moreover, convergence insufficiency is more common in Parkinson’s disease patients. Medication can also influence convergence disorders by exacerbating the symptoms, like antidepressants, through their anticholinergic action on accommodation. Patients usually present with diplopia, asthenopia, headache, letters that “mix together” after sustained near tasks (reading, writing, computer working) and making eye contact. Examination will reveal high exophoria at near. Symptoms cease after resting periods but return with near activities. Treatment of convergence insufficiency aims to improve convergence thought exercises by pencil push-ups therapy, changing fixation from distance to near, stereograms or computer orthoptics. In addition, orthoptic training with prisms or even strabismus surgery is occasionally indicated [**6**].

## Divergence insufficiency

Divergence insufficiency is a disorder present in older adults that manifests itself by horizontal diplopia at distance. Examination will show esodeviation that is comitant in all gazes at distance but profoundly reduced or even presenting exophoria at near. High myopia with long anterior-posterior axis is associated with divergence insufficiency probably due to the anatomical insertion and modified angles of extraocular muscles. Patients usually present with gradual onset, variable horizontal diplopia at distance, asthenopia, and motion sickness. The pattern of sudden onset will redirect the diagnosis towards sixth nerve palsy. Moreover, papilledema and endpoint nystagmus are important in the differential diagnosis of divergence insufficiency pointing the clinician towards finding a neurological disorder. In these cases, neuroimaging should be considered. In many cases, divergence insufficiency will resolve spontaneously. Treatment options include base-out prisms correction for distance that may cause diplopia if they are worn at near, orthoptic exercises and surgery if the misalignment is stable, long-standing and the patients do not tolerate prism glasses.

## Thyroid ophthalmopathy

The most common muscle involved in thyroid-associated ophthalmopathy is the inferior rectus that causes vertical diplopia. This symptom is defined by a chin-up face position. Nevertheless, all the extraocular muscles may be affected in various degrees (in the following decreasing frequency: medial rectus, superior rectus and lateral rectus). For thyroid ophthalmopathy, diplopia is worse in the morning, unlike myasthenia gravis that characteristically has double vision in the second half of the day. 5% of Graves disease patients associate myasthenia gravis. In thyroid ophthalmopathy, exotropia is rare, and should determine the clinician to search for coexisting myasthenia gravis. Pseudo-abducens nerve palsy may be present in medial rectus involvement that will cause abduction restriction [**7**]. Forced duction test will assess the direction of restriction, while orbital imaging (echography, MRI or CT scan) will show enlargement of extraocular muscles. Thyroid function testing is usually normal (TSH, T3, and T4). Autoimmune markers for thyroid dysfunction include thyroid stimulating immunoglobulin (TSI), thyroglobulin antibody, and thyroid peroxidase antibody. Treatment includes thyroid dysfunction therapy, ocular occlusion for diplopia (prismatic lenses are more inconvenient because they require frequent changes), corticosteroid therapy (for acute and severe phases), surgery, radiotherapy. To stop smoking is also very important for therapeutic improvement [**8**].

## Myasthenia gravis

Myasthenia gravis is an autoimmune disease of the neuromuscular junction, mainly found in the older population. Patients complain of uni- or bilateral ptosis and diplopia along with other systemic manifestations (dysphagia, dysarthria, dysphonia, and dyspnea). Symptoms tend to get worse in the second half of the day and improve with rest. The pupil examination is normal and sensory symptoms and pain is usually not present. Myasthenia gravis can make a differential diagnosis with any oculomotor palsy. A simple clinical diagnostic test is the ice test that is highly sensitive and specific for myasthenia. The ice test implies an ice block that is applied on the eyelid for 5 minutes. The test is positive when improvement of ptosis is more than 2 mm. Edrophonium test remains the standard for myasthenia gravis diagnosis and therapy with pyridostigmine and immunosuppressive treatments are necessary [**9**].

## Internuclear ophthalmoplegia

Internuclear ophthalmoplegia is a conjugate lateral gaze disorder. The patients cannot adduct the eye when it looks at contralateral gaze (relative to the affected eye). The unaffected eye abducts but with nystagmus. Symptoms include horizontal diplopia when the eyes are in divergence. This disorder is caused by medial longitudinal fasciculus dysfunction. 

## Postoperative diplopia 

Some patients may develop diplopia after surgical procedures, such as cataract, glaucoma, strabismus, or retinal detachment surgery. For cataract surgery, the causes may be anisometropia more than 3-4 D (cover test indicates orthophoria), astigmatism, trauma of the extraocular muscles after peribulbar injections and myotoxicity induced by the anesthetic agent (that resolves usually in weeks or develops towards restrictive strabismus) and IOL malposition. Binocular diplopia may develop after glaucoma surgery that uses implants, especially Baerveldt implants (the 350 Baerveldt model has most cases) and much less frequent for Ahmed valve or trabeculectomy. The use of scleral buckle for retinal detachment may cause diplopia due to injury of the extraocular muscles. Diplopia can be present following strabismus surgery in patients who are overcorrected. Younger patients can suppress the deviated eye unlike adults or if the suppression was before surgery, they may not be able to shift it [**10**].

## Treatment of diplopia

The management of diplopia must be according to the cause that determined it. Common sense will indicate the treatment of the pathology that caused it whenever this is possible. Symptomatic treatment is often used in patients with diplopia who have a significant morbidity rate associated with loss of orientation and confusion. The goal in these patients is to regain single binocular vision. Unilateral occlusion of the poorer eye may be used as treatment when other measures are not successful. In younger patients, the occlusion should be alternative because they have the risk of developing amblyopia. In this case, options include eye patch, frosting of eyeglass lens or semi-opaque tape and contact lens with opaque center. Temporary monocular occlusion is usually necessary in patients who have diplopia as a cause of ischemic cranial nerve palsies, until diplopia resolves by itself. Fresnel prisms for the realignment of visual axis may also be used in case of vertical and horizontal diplopia but not in torsional diplopia or in the resolving stages, when ocular misalignment may frequently change. Surgical treatment of strabismus is indicated in long-standing, unchanged misalignment for more than 12 months, when other therapies have not been successful. Another option includes botulinum injection in the antagonist of the paralyzed muscle with an effect of 3 to 6 months for a single dose [**11**].
